# Supplementary material for: Are carbon emissions trading and green financial instruments synergistic? -Comprehensive quantitative research based on content analysis
Source: PLoS One. 2024 Mar 7;19(3):e0298601. doi: 10.1371/journal.pone.0298601 (PMC10919601; doi:10.1371/journal.pone.0298601)
Supplement: S1 Data — (ZIP) [file pone.0298601.s001.zip › Data/地级市Z_F工作报告.zip!/║╙▒▒/╟╪╗╩╡║╩╨/└┤╫╘╣1⁄2╓┌║┼í╛┬φ┐╦╩2╛▌═°í┐_2015.docx]

各位代表：

现在，我代表秦皇岛市人民政府向大会作工作报告，请予审议，并请各位政协委员和列席会议的同志提出意见。

一、2014年工作回顾

过去的一年，面对复杂严峻的宏观形势，我们在省委、省政府和市委的坚强领导下，以开展党的群众路线教育实践活动为动力，深入实施开放强市、产业立市、旅游兴市、文化铸市战略，认真组织项目建设落实年、环境整治攻坚年、基层工作服务年行动，着力稳增长、调结构、促改革、治污染、惠民生，努力实现市十三届人大二次会议确定的目标任务。全年实现地区生产总值1200亿元，比上年增长5%；全部财政收入206.4亿元，一般公共预算收入113.7亿元，分别增长4.5%和3.8%；全社会固定资产投资808.7亿元，增长2.8%；社会消费品零售总额571.3亿元，增长12.2%；预计城镇居民人均可支配收入26231元，农村居民人均可支配收入10016元，分别增长9.2%和11.2%，均明显高于经济增速。

(一)促进经济稳定增长，经济质量效益得到提高。坚持扩总量和优结构并举，深入开展央企、知名民企、外企入秦等活动，实施省市重点项目87个，完成投资187.4亿元。东扩港区建设正式启动，150航道立项审批等西港搬迁改造提升前期工作基本完成；中信戴卡凯斯曼、际华军民产业园等项目投产或相继竣工。全年实际利用外资8.1亿美元，引进内资412.7亿元，分别增长10%和19.9%。三次产业协调发展。主要农产品产量稳定增长，农业产业化经营率全省领先。规模以上工业实现增加值341.7亿元、利润28.5亿元，分别增长4.7%和10.9%。规模以上工业增加值中，装备制造、高新技术产业占比分别达到33.8%和30.8%，电子信息制造业占比超过玻璃工业，达到4.5%。服务业对经济发展贡献率达51.6%。接待中外游客2852万人次，实现旅游总收入293.6亿元，分别增长9.9%和14.6%，游客人均消费突破1000元。港口货物吞吐量2.74亿吨，集装箱41.4万标箱。出口总额28.5亿美元，增长18%。河北银行秦皇岛分行正式运营。我市被列为全国养老服务业综合改革试点市。

(二)积极主动对接京津，务实合作取得明显进展。把握京津冀协同发展机遇，明确“一都三区一片”发展定位，积极对接、主动合作，着力承接首都功能疏解、京津产业转移和要素外溢。中关村海淀园秦皇岛分园揭牌开园，千方科技等7家企业入驻；与中建材、顺鑫集团等签订了合作协议；北京化工大学秦皇岛分校等25个项目正开展前期工作；北斗卫星数据中心等13个项目已开工建设；中兴网信智慧城市北方基地等项目落户数据产业基地，智慧城市一期项目上线运行。借力暑期发展自己，沿海基干林生态保护、北戴河第二快速通道建设等一批项目得到国家支持；京唐秦高铁纳入京津冀交通领域率先突破工作方案。

(三)加快改革创新步伐，发展动力活力进一步增强。下大力简政放权，市级审批事项由425项减少到228项。完成市级层面食品药品监督管理体制改革。农村土地承包经营权确权登记颁证29.5万亩，抚宁石门村、青龙大森店等12个试点村产权制度改革顺利完成。全面实施商事登记制度改革，新增市场主体2.3万户，民营经济占地区生产总值的比重达到67%。新增挂牌上市企业7家。完成工业技改投资180.1亿元，新增省级技术研发机构3家，新认定高新技术企业17家，科技型中小企业达到567家；中科院北京分院等院所院校与我市合作取得积极进展。

(四)扎实推进城乡建设，人居环境不断优化。北戴河机场工程通过竣工验收，沿海高速北戴河联络线、承秦高速石门寨连接线、北环路上跨铁路桥建成通车。北戴河新区污水处理厂开工建设。扎实开展“三治两提”攻坚行动，县城和重点镇环境容貌和管理水平进一步提升，8个镇被列为国家级重点镇。完成136个村的农村面貌改造提升任务，改造农村危房7070户。解决16.79万农村人口饮水安全问题。连接海港区与北戴河区的全国最长的滨海木栈道全线贯通。基本完成北戴河近岸海域综合整治三年行动计划，海水浴场水质全部达标。狠抓大气污染防治，淘汰黄标车4.62万辆，更换新能源公交车484辆，推广秸秆成型燃料炉具1.94万户，压减炼铁产能280万吨、炼钢产能210万吨，工程削减煤炭消费量211.9万吨，全年空气质量优良天数达到239天，比上年增加30天。单位生产总值能耗预计下降4.7%，化学需氧量、二氧化硫、氨氮和氮氧化物排放量分别削减1.5%、1.6%、1.5%和5.5%。人工造林13.4万亩，建成区新增和改扩建绿地160万平方米。

(五)大力发展社会事业，人民生活持续改善。落实“十项惠民举措”，认真办好30件实事。城镇新增就业4.9万人，转移农村劳动力2.16万人，城镇登记失业率3.37%。城镇低保标准提高到每人每月450元以上，农村低保标准提高到每人每年2500元以上，城乡低保有效保障对象11万人。高龄补贴惠及1.7万老年人。提高失独家庭和独生子女伤残家庭特别扶助金标准。养老保险金标准人均每月提高199元，医疗保险实现制度全覆盖，建立城镇居民大病保险制度，在职城镇职工、城镇居民医疗保险报销比例上限分别达到90%和75%，新农合参合率达97.72%。实施农村孕产妇免费产前筛查。新开工保障性住房和棚户区改造安置住房1.1万套。开展扶贫帮困“春雨行动”，3.76万贫困人口实现稳定脱贫。义务教育均衡发展，治理择校成效明显。市精神卫生中心开工建设，县级公立医院改革稳步推进。扎实开展“中国梦·赶考行”活动，创建全国文明城市工作取得积极成效，公共文化服务能力进一步提升。成功举办北戴河科技成果展、秦皇岛国际马拉松赛、“长城情·中国梦”感恩等重大活动 天火大道无弹窗。安全生产、食品药品安全形势稳定向好，刑事和治安发案明显下降。圆满完成暑期服务保障任务。我市被央视评为中国最幸福城市20强。国防动员、民族宗教、新闻广电出版、外事侨务、人民防空、方志档案、气象地震、地理信息、援藏援疆、人口计生、妇女儿童、老龄、慈善、残疾人等事业健康发展。

(六)加强政府自身建设，服务水平全面提升。扎实开展党的群众路线教育实践活动，严格落实中央“八项规定”，“四风”问题得到有效整治，市本级“三公”经费比上年压减20%以上。推行机关标准化建设，健全政府法律顾问制度，规范行政决策机制，依法行政能力进一步加强。自觉接受人大法律监督、工作监督，政协的民主监督，累计办理省市人大代表建议、政协提案561件，按时办复率100%，满意率99%以上。扎实推进反腐倡廉工作，立案903件，查处公职人员868人。

各位代表!过去的一年，我们立足发展实际，狠抓事关当前和长远发展的重大问题，以关键环节的突破带动活跃全局。一是优化发展环境。坚持以开放为统揽，补短板、蓄后劲，促改革、激活力，基础设施日趋完善，市场主体明显增加，区位、生态、暑期等比较优势正加速转化为发展优势、竞争优势。二是转变发展方式。注重速度、结构、质量和效益的高度统一，发挥科技创新的引领作用，努力化解过剩产能，优化产业结构，提升经济运行质量，可持续发展能力明显增强。三是融入协同发展。强化为京津服务的理念，搭建对接平台，创新体制机制，积极推进与京津基础设施互联互通、产业发展对接协作、资源要素双向流动，各级各方面对我市的期望更多、关注更高、支持更大。四是做靓生态品牌。深度推进大气污染防治、近岸海域综合整治，提升城乡绿化水平，生态环境质量和城市宜居水平居全省前列，对内对外吸引力不断增强。

各位代表!过去的一年，在国际形势复杂多变、国内“三期叠加”压力不断加大、经济增长动力青黄不接的背景下，我们发展遇到的困难挑战异常突出，能够取得这样的成绩实属不易。这是省委、省政府和市委正确领导的结果，是市人大、市政协和社会各界有效监督、鼎力支持的结果，是全市上下勠力同心、拼搏进取的结果。在此，我代表市政府，向全市人民，向人大代表、政协委员，向各民主党派、工商联、无党派人士和人民团体，向驻秦部队、武警官兵、政法干警，向中直、省直机关驻秦各单位，向所有关心支持秦皇岛发展的港澳台同胞、海外侨胞和国内外朋友，致以崇高的敬意和衷心的感谢!

回顾过去的一年，我们清醒地认识到，我市经济社会发展长期积累的一些深层次矛盾和问题愈加凸显，经济下行压力加大，去年全市地区生产总值、固定资产投资、规模以上工业增加值、进出口总额、社会消费品零售总额、城镇居民人均可支配收入等指标增速没有完成预期目标。经济总量小，重大支撑项目少，发展后劲不足的问题没有得到明显缓解。传统产业链条短，拉动力不足，新兴产业还没有形成有效支撑，企业创新能力不强，产品附加值较低，发展方式粗放。财政收入增长缓慢，收支矛盾日益加剧，偿债压力巨大。社会事业和公共服务设施建设方面尚有不少欠账。政府工作与人民群众的期盼存在一定差距，一些政府部门和工作人员依法行政和服务意识淡薄、懒政怠政，一些领域和地方消极腐败现象还比较严重。我们将进一步强化措施，下大力解决这些问题，决不辜负全市人民的厚望!二、2015年目标任务

今年是全面深化改革的关键之年，是全面推进法治秦皇岛建设的开局之年，也是全面完成“十二五”规划的收官之年。当前，我国经济发展进入了新常态，虽然面临需求总体偏弱、转型任重道远、潜在风险暴露等多重困难和挑战，但发展速度更趋平稳、动力更为多元、结构更加优化。特别是京津冀协同发展战略、河北沿海地区率先发展战略深入推进，改革开放红利持续释放，为我们发展创造了难得的历史机遇，提供了不竭的动力。我们一定抢抓机遇、主动作为，推动秦皇岛科学发展、绿色崛起，加快打造河北沿海地区率先发展的增长极。

政府工作的总体要求是：认真落实中央、省委省政府和市委重大部署，坚持稳中求进工作总基调，主动适应经济发展新常态，把加快发展作为第一要务，深入实施开放强市、产业立市、旅游兴市、文化铸市战略，着力转方式调结构，着力培育经济增长点，着力开展改革攻坚，着力强化创新驱动，着力改善生态环境，着力保障改善民生，着力推进依法治市，促进经济持续健康发展和社会和谐稳定，开创沿海强市、美丽港城建设新局面。

全年经济社会发展的主要预期目标是：地区生产总值增长7%左右；全部财政收入增长6%，一般公共预算收入增长5%；全社会固定资产投资增长15%；社会消费品零售总额增长11%；进出口总额增长5%，实际利用外资增长6%；单位生产总值能耗下降3%，化学需氧量、二氧化硫、氨氮、氮氧化物排放量分别削减1.2%、1.49%、1.2%和1.31%，细颗粒物(PM2.5)浓度下降5%；城乡居民人均可支配收入均增长10%；居民消费价格指数涨幅控制在3%左右；城镇登记失业率控制在4%以内；人口自然增长率控制在6.8‰以内。提出这样的指标，把握了“稳”和“进”的平衡点、突出了“质”和“量”的增长点，是符合上级要求和我市发展实际的。工作中，力求发展得更快、更好些。

重点抓好八项主要任务：

(一)扭住第一要务不放松，在力促经济稳步增长上取得新成效。既注重长远打基础、扩总量，又注重当前提速度、增效益，加快沿海强市建设步伐。

大力加强项目建设。把项目建设作为稳增长、调结构的关键环节来抓。实施100个重点建设项目，推进100个前期项目，力争臻鼎科技二期等60个项目开工建设，骊骅淀粉综合技改等20个项目竣工投产。着力推进能有效增加就业和税源、符合我市发展定位和环保要求的大项目、好项目，确保重点项目中投资10亿元以上的产业项目不少于50个，产业投资增长20%以上。对2013年以来签约的重点项目，落实“五个一”推进机制，力促早日落地开工。加大项目调度力度和频次，实行严格的考核机制，严格兑现奖惩，营造上大项目、上好项目、快上项目的浓厚氛围。

促进工业提档升级。把提高工业质量效益作为稳增长的现实路径，大力发展高科技、低能耗、无污染的工业项目，特别是终端产品生产项目，力促中粮燃料乙醇等重点项目落地开工，新建成工业项目尽快投产达效，确保规模以上工业增加值增长6%以上。强化工业运行监测服务，加强生产要素保障和市场供需衔接，帮助企业稳定生产、扩大市场、减轻负担。实行重点工业企业服务直通车和市领导分包联系制度，选择100家规上企业和100家规下企业重点扶持，促进高成长性企业快速发展，促进停产、亏损和特困企业扭亏为盈。完善个转企、小升规扶持政策，确保新增规上工业企业70家左右。

强化园区支撑作用。把园区建设作为稳增长的重要载体，着力提高园区投资率、就业率、税收贡献率。推进秦皇岛开发区西扩，主攻高端装备制造业、战略新兴产业和现代生产性服务业，确保秦皇岛能源交易中心等项目取得有效进展。加快北戴河新区开发建设，与昌黎县一体化发展，主攻休闲旅游、康养医疗、会议会展、教育科研等业态，推进恒博华贸网球中心、北京化工大学秦皇岛分校等项目取得明显成效。理顺西部经济开发区体制，延伸产业链条，发展循环经济。推进山海关临港开发区与秦皇岛开发区、秦皇岛临港物流园区与东部循环经济园区合作发展。实施一区多园，采取托管、异地共建等方式，优化滨海地区、平原地区和北部山区生产力布局，合理摆放工业、战略新兴产业和现代服务业项目，吸引社会资本参与园区开发建设。争取在秦皇岛开发区、北戴河新区设立行政审批局。加快北戴河新区、海港区省级高新区建设。

着力提升开放水平。强化开放意识和开放思维，加大招商引资力度，选择重点区域设立委托招商机构，实施以商招商、地区总代理人招商，依托现有产业推进产业链招商，争取一批重大项目签约落地。依托秦皇岛开发区、出口加工区，谋划建设综合保税区，吸引境内外关联企业聚集；推进属地申报、属地放行通关模式，开展商检、海关、金融、保险、代理等“一站式”服务。帮扶水产品、汽车零配件等重点企业扩大出口规模，争取秦皇岛港成为国家进境水果指定口岸。鼓励有条件的企业积极承揽境外工程项目，开展境外技术和投资合作，提升外经外贸规模和水平。

努力释放消费潜力。完善城乡便民服务网络，加强专业市场和茂业综合体等项目建设，发展连锁经营和现代物流等新业态。抓好国家信息消费试点工作，启动实施信息消费产品产业化等十大试点工程，扩大互联网、物联网等信息消费。实施节能产品惠民工程，促进绿色消费。实施养老健康家政、教育文化体育等领域消费工程，支持社会力量举办养老、健身、健康、医疗等服务机构。多渠道增加居民收入，合理提高公务员津补贴和事业单位绩效工资。落实国家、省有关政策，完善针对性措施，努力消化存量、有效控制增量，促进房地产业健康发展。

加快发展是我市经济社会发展的主旋律。我们将牢牢把握促进经济增长的关键环节，千方百计扩总量、提速度，优结构、增效益，让秦皇岛发展的动力更足、后劲更大、实力更强!

(二)加大对接京津工作力度，在融入区域协同发展上争取新突破。全面对接国家、省顶层设计，以有为精神和务实态度，大力推进京津冀协同发展工作，确保取得扎实成效。

健全完善协同发展平台。完善各层次规划，做实做好六大平台。依托北戴河，重点承接首都二、三级行政机构及国家咨询机构、研究机构等单位疏解和转移，打造非首都核心功能承接平台；依托北戴河新区，重点承接京津康养医疗、教育、体育、文化等领域功能和产业转移，打造公共服务功能承接平台；依托北戴河区、北戴河新区和海港区，放大北戴河会议品牌优势，积极承办国际国内大型会议，承接首都金融机构、区域总部等功能转移，打造会议商务功能承接平台；依托秦皇岛港和临港物流园区，加强与北京物流企业合作，承接跨区分拨、城际配送、中转贸易等功能，建设现代物流结算中心和临港物流基地，打造现代物流产业承接平台；依托秦皇岛开发区，重点承接京津高端技术人才、高新技术产业孵化和成果转化功能以及先进制造、电子信息、节能环保等产业转移，打造战略性新兴产业承接平台；依托抚宁县和昌黎县，积极承接京津农产品批发市场和农贸市场外迁转移，打造京津农产品交易功能承接和供应平台。

推进交通一体化进程。重点争取京唐秦城际铁路项目尽快开工建设，早日实现京秦“1小时通达”；积极协助推进高速公路京秦二通道和连接线建设；争取承秦铁路项目纳入国家相关建设规划，促进秦皇岛港与“三北”腹地的互联互通；完成北戴河机场建设后续工作，确保年内转场通航，争取将北戴河机场列入国家口岸开放“十三五”规划。争取利用山海关机场，发展通用航空业务。

积极承接京津产业转移。以中关村海淀园秦皇岛分园为依托，用足用好国家政策，力争北大科技产业园等项目开工。有效推进与顺鑫集团在农副产品加工、农产品物流等方面的合作。加快推进北戴河国际健康城建设，加强与国内重点高校、重点医院的合作，实施市第二医院搬迁等起步区项目建设。密切与京津有关企业、院校院所、机构的联系沟通，加快中科院技术成果转化基地等项目的推进步伐，争取北京科技大学、河北医科大学等高等院校落户北戴河新区，力争一批高端装备制造业、劳动密集型产业的新项目向我市转移。

推进西港搬迁改造提升。把西港搬迁改造提升作为落实京津冀协同发展战略、河北沿海地区率先发展战略的重要抓手，加快建设综合性新港区、临港产业聚集区和滨海新城区。与河北港口集团协作配合，力争秦皇岛港总体规划、秦山地区铁路布局调整和集疏港路网体系方案早日获批，尽快完成物流园区控制性详细规划，搞好西港片区景观规划和大小码头片区景观设计，实施150航道建设，确保东扩港区建设和西港区企业搬迁等工作取得实质进展。推进秦皇岛港转型升级，拓展集装箱和杂货业务，利用西港区现有深水码头谋划改建邮轮母港，提升海上旅游品质。

我们将抓住并利用好京津冀协同发展这一重大机遇，以大北戴河为核心，着眼建设国际旅游休闲目的地、国际贸易交流基地、国际会议会展基地、国际康养基地、国际文化交流和体育赛事基地，把秦皇岛打造成一张靓丽的国际名片，谱写京津冀协同发展的秦皇岛华章!

(三)加快推进农业现代化，在转变农业发展方式上迈出新步伐。坚定不移走特色新型农业现代化道路，稳粮增收、提质增效、创新驱动，促进集约发展、可持续发展。

保障农产品有效供给。实施耕地质量保护和提升行动，推进建设占用耕地剥离耕作层土壤再利用，建设优质水稻生产基地10万亩、优质花生生产基地20万亩。加快中小河流、农田水利等工程建设，解决好农田灌溉“最后一公里”问题 礼拜五秘书网。加强“菜篮子”建设，新增设施蔬菜1万亩、果品基地1.8万亩；推动畜禽养殖向规模化、集约化、标准化转变，抓好抚宁生猪、卢龙肉羊、青龙肉鸡省级标准化示范县建设；推进水产生态养殖基地建设，坚决清理整治“绝户网”，促进渔业健康发展。强化病虫害监测预警和植物疫情防控，开展农作物病虫害专业化统防统治。

发展现代农业。像抓工业园区一样抓现代农业园区建设，推进农村一二三产融合发展，争创国家现代农业示范区。每个县区都要依托资源优势，建设现代农业园区。扶持龙头企业发展，加快正大食品二期、宏都生猪电子交易平台等项目建设，稳步提升农业产业化经营率。推动农业科技进步，完善科技服务体系，引进推广优良品种，提高农机化装备和技术水平。积极发展现代生态循环农业，减少农业投入品过量使用，促进秸秆、畜禽粪便资源化利用和农田残膜回收；推进“三品一标”认证，创建无公害农产品生产基地55万亩、全国绿色食品原料标准化基地16万亩。

深化农村改革。搞好土地承包经营权确权登记，健全农村产权交易市场。制定促进土地承包经营权有序流转激励政策和经营权抵押贷款办法，引导和规范土地承包经营权有序流转，发展多种形式适度规模经营，培育壮大新型农业经营主体。完善农业补贴办法，健全耕地保护补偿、生态效益补偿等机制，加大金融支农服务力度。推进供销社综合改革，构建新型农业社会化服务体系，促进农产品产销衔接，提高农民务农收入。创造条件增加农民财产性收入和转移性收入。推进农村二三产业转型升级和农民创业，转移农村劳动力就业1.7万人，增加农民务工收入。

改善农村面貌。扎实搞好美丽乡村建设，继续实施农村面貌改造提升行动，抓好省级重点村面貌改造提升工作，打造以北戴河片区为核心的精品示范区，扶持建设一批具有历史、地域、民族特色的景观村镇。实施精准扶贫，培育特色致富产业，加快贫困地区脱贫步伐。

农业强则中国强，农村美则中国美，农民富则中国富。我们将坚持把“三农”工作摆在突出位置，推动农业现代化与新型工业化、信息化、城镇化同步发展，努力在农业增效、农民增收、农村繁荣上取得更加令人满意的成效!

(四)加快产业转型升级，在构建现代产业体系上有新作为。坚持无中生有、有中生新，推动优势产业高端化、特色产业集群化、新兴产业规模化，打造新常态下秦皇岛经济升级版。

改造提升传统产业。推动信息化和工业化深度融合，培育示范企业。鼓励引导企业加大技改投入，用高新技术和先进适用技术改造提升传统产业，实施重点技改项目200个。依托首秦、美铝渤海等龙头企业，引进配套延伸项目，促进区域企业协同互动，推动金属压延业向精深加工下游产业发展。依托山船重工、哈电重装、天威秦变等企业，加快发展海洋工程、核电、大型输变电等装备；依托中铁山桥、中信戴卡、方华机械等企业，提高高速铁路设备、汽车零部件研发制造水平，壮大以高端制造、智能制造为重点的装备制造业。实施金海食品大豆蛋白扩建等项目，抓好茅台酒庄等干红产业项目，促进粮油食品业向高端、高质、高效方向发展。

培育发展战略新兴产业。以聚集发展、壮大规模为核心，依托海湾公司、康泰医学等企业，发展楼宇自控、智能传感、电子医疗等产业；以中兴网信智慧城市北方基地为重点，加快秦皇岛数据产业基地建设，力争电子信息产业主营业务收入增长20%以上。加快秦皇岛开发区节能环保产业园区建设，推进阿尔法工业园装配式住宅产业化研发基地等20个节能环保重点项目，促进节能环保产业提速发展。支持博硕光电等企业发展，争取100万兆瓦分布式光伏发电等项目落地，推动北大医疗健康产业基地、华恒生物等项目加快建设，培育新材料、新能源、生物医药等产业。依托临海临港优势，积极发展海洋经济。

做大做优现代服务业。坚持生活性服务业和生产性服务业并重，确保服务业增加值增长8%以上。加快旅游业转型升级，推进圣蓝皇家海洋公园、五佛山森林公园、祖山文化旅游等项目建设，拓展高端旅游客源市场，缓解淡旺季矛盾，提高游客人均消费水平；加强抚宁大新寨、昌黎葡萄沟和卢龙鲍子沟等景区开发，塑造休闲乡村品牌；争取在北戴河新区实行离区免税政策，打造京津冀区域旅游免税购物区。推进国家级煤炭交易中心、国投泰盛大宗商品交易中心、中运临港国际农产品物流基地建设，打造现代物流业发展新高地。加快中豪金山现代新兴服务产业基地建设，壮大服务外包业，培育发展技术服务、银行保险、研发设计等业态。抓好北戴河怪楼文化艺术产业园、山海关古城开发利用、华莱坞文化产业园等项目，发展文化产业，培育知名文化品牌。推进国际会议中心建设，积极承办国际国内高端峰会和高端论坛。开展养老服务业综合改革试点工作，培育集医疗、康复、保健、养生等为一体的高端产业集群。

加快产业转型升级是掌握经济工作主动权，确保经济行稳致远的重要途径。我们将着力构建以战略新兴产业为先导、先进制造业和现代服务业为支撑的现代产业体系，切实增强产业核心竞争力，推动产业规模效益再上一个新台阶!

(五)改革创新、激发活力，在更好地发挥市场在资源配置中的决定性作用和政府作用上取得新进展。以改革求突破，以创新促升级，用好国家服务业综合改革、创新型城市等试点政策，破解体制机制障碍，释放潜在活力，增强发展动力。

强力推进简政放权。进一步取消和下放行政审批事项，向城区下放与项目审批有关的经济管理权限，能放的全部下放，能取消的全部取消；清理行政事业性收费和政府性基金项目，有收费幅度的一律按下限收费。制定行政审批事项目录和管理办法，推进省市县三级行政审批事项网上一体化审批，在建设、规划等领域推行联合审批制。优化建设项目审批流程，强化审批代办制，推行重点项目业主办理手续不接触审批单位、拆迁进地不接触当地群众制度。做好“三证合一”登记制度改革试点工作。健全市县乡村四级政务(便民)服务体系。

深化重点领域改革。深化财税体制改革，按照新《预算法》要求，推行全面规范、公开透明的财政预算制度，实行全过程绩效预算管理；扩大“营改增”范围。推进国资经营监管体系改革，清理、盘活经营性国有资产，发展混合所有制经济。整合市级融资平台。鼓励民间资本发起设立投资基金和股权投资基金，推广政府与社会资本合作(PPP)模式，吸引社会资本投入城市基础设施、公共服务领域。设立2.5亿元股权投资基金，放大投资规模，引导投资向科技含量高、绿色循环低碳、现代服务业等产业项目倾斜。积极推进金融服务创新，稳步推进小额贷款公司试点工作，支持中小微企业和农业新型经营主体发展壮大。加强金融监督管理，有效防范和化解各类债务金融风险。稳步推进医药卫生、工商质监行政管理体制、机关事业单位养老保险及公车等改革，抓好水电气等资源性产品价格改革。

激发全民创业热情。完善扶持政策，改善创业环境，重商、亲商、扶商、兴商，营造每个人都有创业机会的浓厚氛围，促进民营经济大发展快发展。实施全民创业行动，加快创业孵化基地、大学生创业园、高层次人才创业园等平台建设。鼓励下岗职工再就业再创业、高校毕业生创新创业、复员转业军人自主择业、农村转移劳动力返乡创业。坚持非禁即入、非禁即准，清理不合理规定和隐性壁垒，鼓励民间资本进入交通、能源、水利、生态、医疗等领域。搭建政银企保对接合作平台，加大对成长型、潜力型民营企业的信贷支持力度。鼓励企业通过上市挂牌、发行债券等方式筹集资金，每个县区每年都有新企业挂牌上市。

强化创新体系建设。把科技创新作为建设创新型城市的中心任务来抓，搞好政产学研结合，促进更多科技成果转化为生产力，努力打造中国北方科技城、创新城。发挥企业创新主体作用，鼓励和支持骨干企业联合上下游企业组建产业技术创新联盟，拓展与高等院校、科研院所的对接合作，建设院士工作站、博士后工作站，力争科技小巨人企业达到20家，高新技术企业达到92家，工程技术研究中心、重点实验室、企业技术中心等研发机构达到110家。强化政府创新主导作用，充分利用燕山大学等本地高校和科研院所资源，扩展科技创新服务中心功能，提供社会化、专业化服务；抓好清华大学、天津大学等产业研究院建设，打造全省沿海创新创业人才高地。落实省改革科技成果类无形资产处置方式、深化科技成果转化收益分配改革等10条政策，调动科技人员创新积极性。依托北戴河科技成果展，推进科技成果交易中心建设，搭建科技成果在我市转化的常态化平台。加强知识产权保护，探索开展专利权、科技成果投资入股试点工作。

改革创新永无止境。我们将加快释放改革开放红利，为民营企业等各类市场主体创造更为平等、公平竞争的市场环境，让港城儿女的聪明才智在大众创业、万众创新中充分涌流!(六)统筹城乡一体化发展，在推进新型城镇化建设上实现新提升。坚持以人为本，统筹规划、综合推进、重点突破，提升新型城镇化质量水平，创建全国文明城市。

提升城乡综合承载能力。适应发展需求，谋划启动城市总体规划修编，深化完善专项规划，建立健全控制性详细规划动态维护机制。适时推进行政区划调整。坚持土地节约、集约利用原则，加强土地征收、出让、利用和批后监管。统筹电力、通讯、给排水等地下管网管廊建设，促进城市地下空间合理利用。加快建设北戴河第二快速通道，实施西外环路、102国道卢龙至抚宁段、出海路石门寨至杜庄段等12项道路修建工程，推进北戴河机场快速通道、102国道市区段绕城通道、卢龙至北戴河新区高速支线建设，打造市域半小时交通圈。开工建设引青三期项目，实施石河水库清淤工程。务实推进与华润燃气合作，提升居民用气保障能力。积极引入社会资本参与北戴河西部水厂建设。加快北戴河新区等3个污水处理厂建设，提升公共设施整体水平。

抓好城市环境综合治理。实施街景整治提升工程，完成海港区西环路、山海关关城南路、北戴河草厂西路等25条城市区街道整治提升任务。用好国家开发银行贷款，推进归提寨片区、秦皇岛火车站周边片区、海阳片区、东李庄片区等老城区、城乡接合部的整体改造。抓好老旧小区、背街小巷环境治理，整治占道经营、市场外溢、三轮车非法上路、露天烧烤、噪音污染等问题。加强城乡无障碍环境建设。有序推进智慧城市建设，智慧旅游、智慧医疗、智慧交通、平安城市等一期项目建成使用，启动智慧社区、智慧政务、智慧安监、智慧环保等二期项目，提高城市管理数字化、精细化水平。抓好城市社区规范化建设，提升管理水平。

加强县城和小城镇建设。完善县域城乡规划体系，促进“多规合一”，推进昌黎、抚宁县城与主城区同城化发展，基础设施建设与主城区接轨；凸显卢龙、青龙县城历史文化内涵，培育宜居宜业的卫星城市。抓好县城建设攻坚，深入开展“三治两提”攻坚行动，搞好水、电、路以及垃圾、污水处理等基础设施建设，增强承载力和聚集力。以13个国家、省级重点镇为重点，适度下放管理职能和权限，培育经济发达、环境优美、功能完善、特色鲜明的小城镇。加强与天津东丽区合作，加快薛家营城镇化示范区建设。

着力增强县域经济实力。创新县域经济发展推进机制，加强分类指导，生产要素向县域项目和园区倾斜，加快改造提升县域钢铁、机械加工、轻工食品等传统产业，发展装备制造、新型建材、新能源等新的支柱产业，促进卓达新型建材园、英利光伏发电、华润风电等一批重点项目早日投产。依托县域葡萄酒、皮毛交易、甘薯粉丝、绒山羊等特色产业，积极引进扶持一批产业链上下延伸项目，打造知名品牌，提升在全省、全国的影响力。

新型城镇化是扩内需、稳增长、转方式的潜力所在。我们将遵循规律、因地制宜、稳妥推进，努力走出一条符合市情实际的新型城镇化道路，为加快发展提供不竭的动力支撑!

(七)持续加强生态环境建设，在打造全国生态文明先行区上展现新形象。坚持生态文明优先，尊重自然、顺应自然、保护自然，加快建设美丽港城。

抓好大气污染防治。推进集中供热、“煤改气”、“煤改电”，利用微煤雾化技术改造全市城乡燃煤锅炉，以北戴河为试点在全市率先推开。深入开展钢铁、电力、水泥、玻璃等行业大气污染防治攻坚行动，加快脱硫、脱硝和除尘改造。狠抓扬尘污染治理，以朱各庄镇、石门镇、杜庄镇、石门寨镇等区域为重点，完成所有石矿、碎石企业、石灰窑、砖厂等整治任务。减少机动车污染排放，全部淘汰黄标车，在公交、出租车行业推广新能源汽车。

加强水生态建设。巩固北戴河近岸海域环境综合整治成果，突出重点入海河流，抓好工业点源和农业面源污染治理，促进海水质量持续好转。抓好生态水系建设，力争将水源地生态修复、水源涵养、供水能力和河道生态建设等项目，纳入国家国土江河综合整治试点范围。加强水源地环境监测和保护，有效治理水土流失，做好湿地保护工作。强化对排放重金属和其他有毒有害污染物工业企业的监督检查，消除环境安全隐患。

持续推进节能减排。加强工业、交通、建筑等重点领域节能，推行合同能源管理，全市域所有新建建筑执行绿色建筑节能标准。实施新的排污费征收标准，加强主要污染物总量控制。做好低碳城市试点工作，搞好节水、节电、节材活动。开展环境污染第三方治理和碳排放交易试点。提高企业环保、节能、技术准入门槛，严格执行能耗限额、污染物排放特别限制标准，以及差别电价、水价政策。强化“网格化”环境监管，对各类环境违法行为“零容忍”，让非法排污付出更大代价。

掀起造林绿化新高潮。依托“三北”防护林、沿海基干林带改造提升等工程，以河流沿线、高速公路、沿海公路两侧及生态脆弱区为重点，开展大规模造林绿化。实施城区重要道路节点绿化塑景、老旧小区增绿、城区立体绿化等工程。抓好北戴河国家湿地公园及周边生态修复续建工程，确保顺利通过国家验收。积极开展认种认养、种植纪念林等活动，严厉打击盗采乱伐，严防森林火灾。全年新增绿地100万平方米，人工造林10万亩以上，森林覆盖率达到45%。

绿水青山就是我们的金山银山。我们必须尽到我们这一代人的历史责任，像爱护自己的眼睛那样呵护好我们的家园，让青山绿水长留、碧海金沙长在、蓝天白云长驻!

(八)全心全意为人民谋福祉，在保障改善民生和发展社会事业上取得新成果。按照保基本、兜底线、促公平的要求，加快以改善民生为重点的社会建设，尽心竭力让发展成果更多更公平地惠及全市人民。

推动实现更高质量就业。全面落实更加积极的就业政策，完善城乡均等的就业服务体系，千方百计扩大就业，统筹解决好高校毕业生、农村转移劳动力、退役军人等重点人群就业问题。用足用好援企稳岗政策，对依法缴纳各项社会保险的困难企业进行补贴，帮助企业稳定就业岗位。积极开发公益性就业岗位，对零就业家庭、残疾人、低保对象进行分类帮扶。做好职业技能培训工作。加大劳动监察执法力度，有效保障劳动者合法权益。全年城镇新增就业4.5万人。

推进社会保障扩面提质。建立完善社会保障待遇调整机制，探索社会保障多险合一的管理模式。健全完善企业养老保险制度，确保企业退休人员社会化管理服务率保持在90%以上。提高城镇居民医保补助和新农合标准，新农合参合率保持在95%以上。推进城乡一体化养老服务体系建设。抓好保障性安居工程，健全住房保障体系。

全面发展社会事业。办好人民满意的教育，组织实施第二期学前教育三年行动计划，全面改善贫困地区义务教育薄弱学校办学条件，促进义务教育均衡发展；多样化发展普通高中教育，支持职业院校创建国家骨干院校，促进高等教育发展；加强教师队伍建设，全面提高教师素质。巩固完善基本药物制度和基层综合改革成果，推进医疗资源布局优化和医疗卫生基础设施建设，完成市精神卫生中心主体工程，开展百姓放心乡镇卫生院建设。加强基层文化服务队伍建设，完善现代公共文化服务体系，打造一批文化艺术精品。大力发展体育事业，办好第二届马拉松赛等重要赛事，做好河北中基足球俱乐部主场服务保障工作。加强公民思想道德建设，提高城市文明程度。

维护社会安全稳定。开展食品药品安全专项整治，严厉打击制假售假等违法犯罪行为，努力创建食品药品安全放心城市。强化安全监管，力争全年不发生重大以上安全生产事故。搞好法治宣传教育，严厉打击各类违法犯罪行为，建设平安秦皇岛。高度重视人民调解工作，积极化解特殊疑难信访问题，提高信访按时办结率和息诉罢访率。严格程序标准，严肃换届纪律，严抓责任落实，有序做好村委会换届选举工作。扎实做好服务暑期和借力暑期工作，确保实现安全、和谐、简朴、满意的目标。加强国防教育和国防后备力量建设，支持驻秦人民解放军、武警部队全面加强革命化、现代化、正规化建设，推进军民融合式发展，争创全国双拥模范城“六连冠”。支持工会、共青团、妇联等人民团体发挥桥梁纽带作用，做好新闻广电出版、民族宗教、外事侨务、人民防空、气象地震、防灾减灾、地理信息、邮政通信、方志档案、援藏援疆和老龄、慈善、妇女儿童、人口计生等工作。做好“十三五”规划编制工作。

各位代表!尽管当前我市财政收支矛盾突出，但我们要克服困难，千方百计筹措资金，保障民生支出，办好10件为民实事。一是企业退休人员基本养老金提高10%，城乡居民基础养老金由60元提高到75元。二是实施医疗保险市级统筹，实现95万参保人员全市域看病就医“一卡通”；达到规定缴费年限的退休人员，不再缴纳基本医疗保险费，终身享受基本医疗保险待遇。三是解决12万农村人口饮水安全问题，确保全市所有农村居民都能喝上干净水；减免城市1900个特困户和低保户热费。四是开工建设保障性住房和棚户区改造住房10776套，竣工9000套，分配入住9000套；改造农村危房7000套。五是拆除严重影响城区道路通行的地方小铁路；改造河北大街与西外环交叉路口；优化交通信号联动控制系统，缓解交通拥堵；加强停车场规范化管理；县城20公里范围内的农村客运线路公交化运行率达到30%以上 昆仑小说。六是维修改造市区21条街巷路，建设改造路灯700基；取缔城区内燃煤锅炉200台；对老旧供热管网进行更新改造；依法规范住宅小区物业管理。七是改造提升北戴河区剑秋路、七桥、东二路、浪淘沙园、老虎石海上公园和北戴河新区金沙湾沙雕大世界景区等滨海浴场。八是开工建设市民政事业服务中心，建设8所社区居家养老服务中心；实施贫困残疾儿童免费抢救性康复工程，救助680名各类贫困残疾儿童。九是为农村地区中小学学生解决冬季用热水问题；开工建设市博物馆、美术馆；免费向群众开放“三馆一站一室一屋”，推动公共文化资源向社会开放共享；为每个行政村每月放映1场公益电影；为100个村(社区)安装室外体育健身器材。十是建设秦皇岛食品药品检测中心；实现药品生产销售全过程电子监控，力争婴幼儿乳粉、生鲜农产品、保健食品等实现电子追溯；病害猪无害化处理率达到100%。

增进民生福祉、促进社会公平，是一切工作的出发点和落脚点。我们将更加重视社会事业发展，更大力度完善社会保障体系，努力让人民群众在秦皇岛生活得更加幸福、更加自豪、更加美好!

三、全面加强法治政府建设

着眼维护宪法法律权威，大力推进依法行政，切实提升各级政府和政府各部门运用法治思维和法治方式解决问题的能力，加快建设职能科学、权责法定、执法严明、公开公正、廉洁高效、守法诚信的法治政府。

依法履行政府职能。坚持法定职责必须为、法无授权不可为，建立健全权力清单、责任清单和负面清单制度。全面完成市县两级政府权力清单编制及公开工作，推进各级政府机构、职能、权限、程序、责任法定化。

健全依法决策机制。完善公众参与、专家论证、风险评估、合法性审查、集体讨论决定等重大行政决策程序，全面推行政府法律顾问制度、重大决策终身责任追究制度和责任倒查机制。完善重大事项向人大报告制度，积极采纳人大、政协建议，吸取各民主党派、工商联、无党派人士意见，发挥专家学者、老领导、老同志和各类智库的作用。

严格公正文明执法。积极推进综合执法，减少市县两级政府执法队伍种类。推行城市管理领域相对集中行政处罚权。完善执法程序，细化、量化行政裁量标准。落实行政执法责任制，完善纠错问责机制，加强改进行政复议和行政应诉工作，严肃追究违纪违法行政工作人员的责任。加大食品药品、安全生产、环境保护、征地拆迁等关系群众切身利益的重点领域执法力度，维护群众合法利益。

加强行政权力监督。自觉接受人大法律监督、工作监督和政协的民主监督，虚心接受社会监督和舆论监督。加强对政府内部权力的制约，对权力集中的部门和岗位，强化内部流程控制，防止权力滥用。健全各级政府法制机构，强化政府内部层级监督。推进政务“五公开”，加快建设阳光政府。

推进勤政廉政建设。巩固党的群众路线教育实践活动成果，牢记“三严三实”，始终保持昂扬向上、奋发有为的精神状态，崇尚实干、狠抓落实，把各项工作任务项目化、具体化、责任化，确保件件有人抓、事事有人管、层层有责任，让抓落实成为一种常态。严格落实中央“八项规定”，坚持不懈反对“四风”，坚决整治庸政、懒政、怠政问题。牢固树立过紧日子思想，集中财力办大事，严格控制一般性支出，确保“三公”经费只减不增。不折不扣落实党风廉政建设“一岗双责”，严守“底线”、不触“红线”、筑牢“防线”，坚决守好政治纪律和政治规矩。坚持“一案双查”，强化责任追究，对违法违纪行为一查到底、绝不姑息，营造不敢腐、不能腐、不想腐的氛围。

法治政府建设事关改革发展稳定大局，事关人民群众切身利益。只要我们全面加快法治政府建设，提高公务员队伍能力素质，增强政府公信力、执行力、服务力，就一定能够一步一个脚印地把各项事业不断推向前进，一定能够更好地推动秦皇岛转型发展、创新发展、绿色发展、协同发展，一定能够赢得全市人民群众的充分信任和大力支持!

各位代表!党的重托、人民的期待，是我们做好工作的巨大动力，也是我们肩负的重大责任。让我们紧密团结在以习近平同志为总书记的党中央周围，在省委、省政府和市委的坚强领导下，团结拼搏、锐意进取，适应新常态，实现新作为，创造新业绩，为建设沿海强市、美丽港城而努力奋斗!
